# Characterization and Spatial Mapping of the Human Gut Metasecretome

**DOI:** 10.1128/msystems.00717-22

**Published:** 2022-12-05

**Authors:** Florencia Velez-Cortes, Harris Wang

**Affiliations:** a Department of Systems Biology, Columbia University, New York, New York, USA; b Integrated Program in Cellular, Molecular, and Biomedical Studies, Columbia University, New York, New York, USA; c Department of Pathology and Cell Biology, Columbia University, New York, New York, USA; Cleveland Clinic

**Keywords:** gut microbiome, human microbiome, metagenomics

## Abstract

Bacterially secreted proteins play an important role in microbial physiology and ecology in many environments, including the mammalian gut. While gut microbes have been extensively studied over the past decades, little is known about the proteins that they secrete into the gastrointestinal tract. In this study, we developed and applied a computational pipeline to a comprehensive catalog of human-associated metagenome-assembled genomes in order to predict and analyze the bacterial metasecretome of the human gut, i.e., the collection of proteins secreted out of the cytoplasm by human gut bacteria. We identified the presence of large and diverse families of secreted carbohydrate-active enzymes and assessed their phylogenetic distributions across different taxonomic groups, which revealed an enrichment in *Bacteroidetes* and *Verrucomicrobia*. By mapping secreted proteins to available metagenomic data from endoscopic sampling of the human gastrointestinal tract, we specifically pinpointed regions in the upper and lower intestinal tract along the lumen and mucosa where specific glycosidases are secreted by resident microbes. The metasecretome analyzed in this study constitutes the most comprehensive list of secreted proteins produced by human gut bacteria reported to date and serves as a useful resource for the microbiome research community.

**IMPORTANCE** Bacterially secreted proteins are necessary for the proper functioning of bacterial cells and communities. Secreted proteins provide bacterial cells with the ability to harvest resources from the exterior, import these resources into the cell, and signal to other bacteria. In the human gut microbiome, these actions impact host health and allow the maintenance of a healthy gut bacterial community. We utilized computational tools to identify the major components of human gut bacterially secreted proteins and determined their spatial distribution in the gastrointestinal tract. Our analysis of human gut bacterial secreted proteins will allow a better understanding of the impact of gut bacteria on human health and represents a step toward identifying new protein functions with interesting applications in biomedicine and industry.

## INTRODUCTION

The gut microbiome plays a vital role in human metabolism, and its deviation from homeostasis has increasingly been linked to various diseases (1–3). Our understanding of the healthy equilibrium state of the gut microbiome is complicated by the fact that closely related taxa possess vastly different, often understudied, metabolic abilities (4). In particular, gut microbes harbor huge metabolic capacities for biotransformation and degradation of dietary substrates that are otherwise indigestible by the host (5). For instance, bacterial carbohydrate-active enzymes (CAZymes) that process complex dietary and host-derived polysaccharides are abundantly found in the gut microbiome (3, 6, 7). CAZymes not only help convert and release various sugars into absorbable forms for the host, but they also facilitate interspecies cross-feeding (8). Since gut microbes are heterogeneously distributed along the gastrointestinal (GI) tract, their associated metabolic capacities can impact dietary metabolism from the proximal to the distal regions (9). Unfortunately, the biodistributions of bacterial metabolic enzymes across the GI tract and between the luminal and mucosal areas have not been adequately described to date. A deeper understanding of the spatial geography of microbial metabolism can help elucidate key gut metabolic biotransformation processes with relevance for nutrition and human health.

At a cellular resolution, microbially associated metabolism in the gut occurs either in the intracellular compartments of individual bacteria or in the extracellular milieu along the lumen or mucosal interfaces through bacterial secretion of digestive enzymes and proteins. Bacterial secretion mainly takes place through the general secretory (Sec) pathway, which relies on recognition of a N-terminal signal peptide tag on a target protein for active transport across a SecYEG channel (10) out of the cytoplasm. These Sec-exported proteins remain in the periplasmic space, are embedded into the inner or outer membranes, or are completely secreted extracellularly. Gram-negative gut microbes such as *Bacteroidetes* often contain many glycoside hydrolases and polysaccharide lyases, with some genomes encoding hundreds of such CAZymes (5). These CAZymes can often contain secretion-associated peptide sequences (5), which suggests that they may function in the extracellular compartment with community-wide effects (11, 12). Delineating the microbiome secretome can help elucidate the main modulators of bacterial community structure in the gut.

Past studies of protein secretion relied heavily on low-throughput experimental strategies that required expression, purification, and mass spectrometry analysis of the secreted proteins individually (13). More recently, advances in machine learning and protein structure predictions have led to *in silico* predictions of secreted proteins, and this method has been applied on large swaths of genomic data in bacteria from a variety of different environments (12, 14). One study revealed that host-associated bacteria encoded more extracellular proteins than bacteria from other environments (12). However, that study used inferred protein annotations from mapping 16S rRNA amplification data sets to reference genomes, which is an approach that can be limited when trying to annotate protein repertoires that are less conserved. The recent increase in metagenomic data sets and new assembly and binning pipeline improvements have created a wealth of metagenomically assembled genomes (MAGs) that have increased the number and diversity of available bacterial genomes (4, 15, 16). These advances can help better dissect the gut microbial secretome but have not been implemented to date.

Here, we describe a systematic analysis of the human gut secretome using a combination of *in silico* approaches to predict secreted proteins from MAGs and map their spatial distribution along the gastrointestinal tract. We annotated the function of secreted proteins and cataloged their enrichment in specific bacterial taxa in the gut. Analysis of the biogeography of secreted enzymes revealed interesting patterns of distributions that suggested functional specialization in different GI compartments, especially those belonging to CAZymes. This work represents the first large-scale systematic study of secreted proteins in bacterial MAGs associated with the human gut and provides a foundation to facilitate future efforts in gut microbiome manipulation and engineering.

## RESULTS

### Establishing a comprehensive gut bacterial secretome.

**FIG 1:**
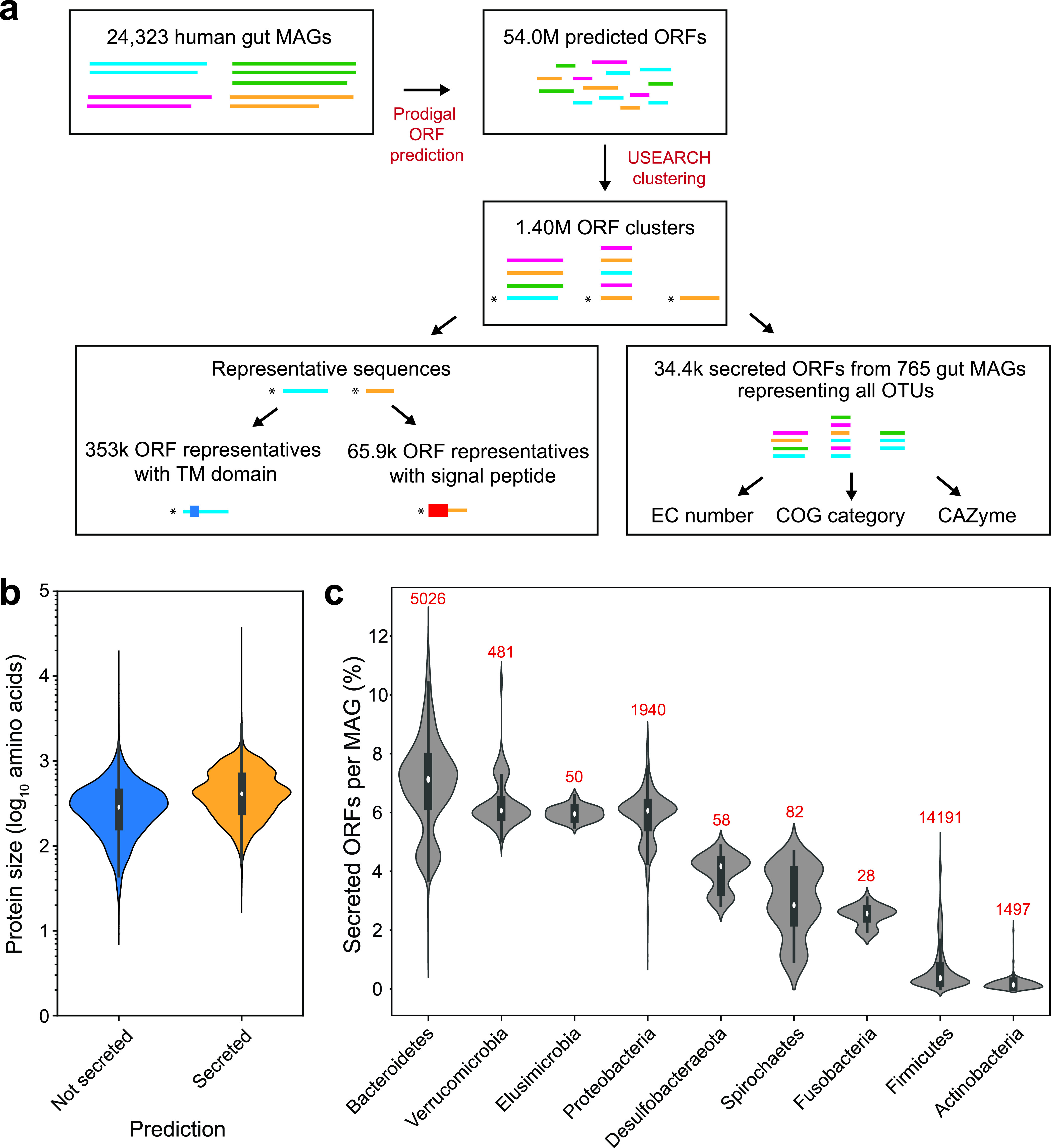
Human gut metasecretome prediction. (a) Illustration of human gut metasecretome prediction pipeline. Centroid sequences are denoted by asterisks. (b) Protein lengths of secreted and nonsecreted proteins. (c) Percentages of secreted ORFs by phylum in HGM MAGs. The numbers of MAGs in each phylum are shown above each violin plot.

We aimed to generate a comprehensive database of secreted proteins (i.e., the secretome) from the human gut microbiota (Fig. 1a) to elucidate their role in intermicrobial and host interactions and explore their functional significance in human physiology and metabolism. We first amassed and annotated 24,323 publicly available high-quality human gut MAGS (15) using Prodigal v2.6.3 (17) (see Materials and Methods), which resulted in 54 million open reading frames (ORFs) that were then clustered at 95% amino acid identity using USEARCH (18) into 1.40 million ORF clusters, each with at least 5 ORF sequences. We utilized a strategy of clustering sequences at a high sequence identity and annotating only representative centroid sequences to reduce the computational resources necessary to analyze the large HGM data set. Thus, the representative centroid sequence of each ORF cluster derived from USEARCH was then annotated using SignalP 5.0 (19) to identify possible presence of signal peptides. Since we are interested only in extracellularly secreted proteins, we filtered out sequences with lipoprotein signal peptides (a SignalP output) because they are likely to be embedded in the membrane (20), and we only maintained those with Sec1 and Tat pathway signal peptides (21). We further used TMHMM v2.0 (22) to identify and exclude sequences with transmembrane domains. A total of 37,511 representative centroid sequences were collated to yield a set of representative secretome ORFs that contained signal peptides but no transmembrane domains or lipoprotein signal peptides. Secretome designation of members within ORF clusters was assigned based on the representative secretome ORFs, which resulted in 1,627,958 ORFs that are putatively secreted out of the cytoplasm, which we designated the “gut bacterial secretome.”

Since the gut is a highly competitive environment, there are likely important evolutionary drivers for optimization of secreted proteins. Secreted proteins are generally encoded with amino acids that are less expensive to produce (23), suggesting that there is a balance between the beneficial and altruistic functions of secreted proteins and their fitness burden on the producing bacteria. We therefore first explored whether there was a correlation between secretion status and protein size. Interestingly, we found that the gut-secreted proteins tend to be larger than nonsecreted proteins from gut bacteria (482 versus 329 amino acids on average, respectively; Mann-Whitney U-test *P* < 10^−3^) (Fig. 1b). This suggested that, at the global level, the benefits of the secreted protein outweigh any metabolic cost of production for the secreting bacteria. When we analyzed the biosynthetic cost of secreted proteins, we observed that *Bacteroidetes* and verrucomicrobial MAGs tended to have similar biosynthesis costs per residue for secreted and nonsecreted proteins, while other gut phyla had a lower median cost for secreted proteins (Kruskal-Wallis H-test, *P* < 10^−56^; Mann-Whitney U test with Bonferroni correction, *P* < 10^−4^) (see Fig. S1 in the supplemental material).

10.1128/msystems.00717-22.1 FIG S1 Difference in biosynthesis cost of secreted and nonsecreted proteins in each phylum with at least 10 representative MAGs. Dashed line indicates that biosynthetic cost of secreted proteins is equal to that of nonsecreted proteins. Download FIG S1, EPS file, 0.8 MB. Copyright © 2022 Velez-Cortes and Wang. 2022 Velez-Cortes and Wang. https://creativecommons.org/licenses/by/4.0/ This content is distributed under the terms of the Creative Commons Attribution 4.0 International license.

*Bacteroidetes* strains, as well as some *Verrucomicrobia*, have been predicted to secrete a large proportion of their proteome (12, 24, 25). To examine the contribution of each phylum to the gut metasecretome, we compared the number of encoded proteins with signal peptides in each of the most prominent and diverse phyla in the human gut, selecting phyla that had at least 5 MAGs in the data set (Fig. 1c). *Bacteroidetes* and *Verrucomicrobia* tended to secrete a larger percentage of their proteome compared to other major gut phyla (Kruskal-Wallis H-test, *P* < 10^−3^; Mann-Whitney *U* test with Bonferroni correction, *P* < 10^−3^), with *Bacteroidetes* the top secreting phylum in the gut (Mann-Whitney *U* test with Bonferroni correction, *P* < 10^−3^). Taken together, these results suggest that *Bacteroidetes* and *Verrucomicrobia* invest significantly in their secreted proteome and are key players in the final composition of the gut bacterial metasecretome.

### Functional assessment of the gut bacterial secretome.

**FIG 2:**
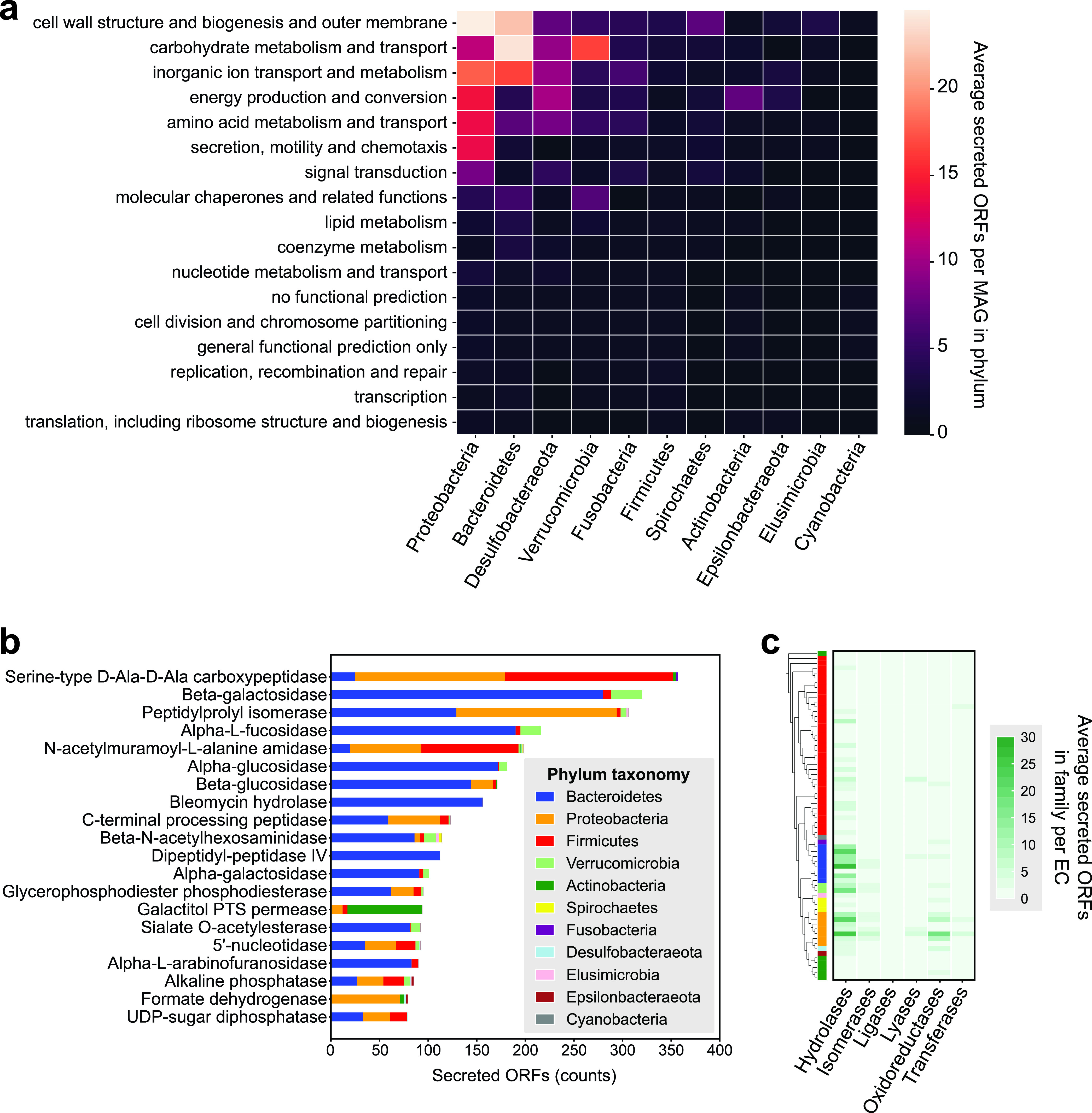
Main functions of the human gut metasecretome. (a) Average number of gut secreted ORFs in each COG category. (b) Top EC annotations of gut secreted proteins. (c) Average numbers of secreted ORFs belonging to each EC category in each MAG, clustered by phylogeny (see Materials and Methods for details).

To survey the functions of the secretome, we used the eggNOG protein ortholog database (26) to annotate secreted ORFs from clusters of proteins with more than 5 member sequences (Table S1). We only annotated one MAG per strain-level operational taxonomic unit (OTU) in the Human Gut Metagenomes (HGM) data set (15), so as to reduce redundancy and limit the computational resources required to do this task. The number of ORFs secreted per MAG was averaged over all representative MAGs in each phylum. The main COG categories among predicted secreted proteins in the gut microbiome were the following: (i) cell wall structure and biogenesis and outer membrane, (ii) carbohydrate metabolism and transport, (iii) inorganic ion transport and metabolism, (iv) energy production and conversion, (v) amino acid metabolism and transport, and (vi) secretion, motility, and chemotaxis (Fig. 2a). *Bacteroidetes* and *Verrucomicrobia* encoded the highest average number of secreted proteins per MAG in the carbohydrate metabolism and transport category. Top Enzyme Commission (EC) categories in the gut metasecretome included proteinases involved in cell membrane biogenesis, nucleotidases, and a variety of CAZymes, such as galactosidases and glucosidases (Fig. 2b), which implied that the human gut metasecretome is highly functionally diverse.

10.1128/msystems.00717-22.8 TABLE S1 eggNOG annotations for HGM representative MAGs, in Excel format. Download Table S1, XLSX file, 4.0 MB. Copyright © 2022 Velez-Cortes and Wang. 2022 Velez-Cortes and Wang. https://creativecommons.org/licenses/by/4.0/ This content is distributed under the terms of the Creative Commons Attribution 4.0 International license.

To understand the differences in secreted protein functions within and across phyla, we examined the average number of encoded secreted enzymes per MAG in each bacterial family represented in the HGM data set. EC annotations were used to identify the distribution of hydrolases, isomerases, transferases, oxidoreductases, lyases, and ligases in gut bacterial families (Fig. 2c). We found that secreted hydrolases were encoded more commonly in the secretomes of *Bacteroidetes*, *Verrucomicrobia*, and certain *Proteobacteria* families. *Bacteroides* strains are known to form complex cross-feeding networks in the mammalian gut via secreted CAZymes (8), while *Verrucomicrobia* like Akkermansia municiphila are known mucin degraders (27, 28). In line with these observations, *Bacteroidetes* and *Verrucomicrobia* MAGs in our study appeared to secrete more hydrolases than other gut phyla. Conversely, *Proteobacteria* secreted more oxidoreductases than other gut phyla included in this study, which was interesting since there are only a few known oxidoreductases that are extracellular in gut bacteria. One such case is Cgr2, a reductase encoded by Eggerthella lenta gut strains, which has the ability to metabolize and inactivate cardiac drug digoxin (29).

**FIG 3:**
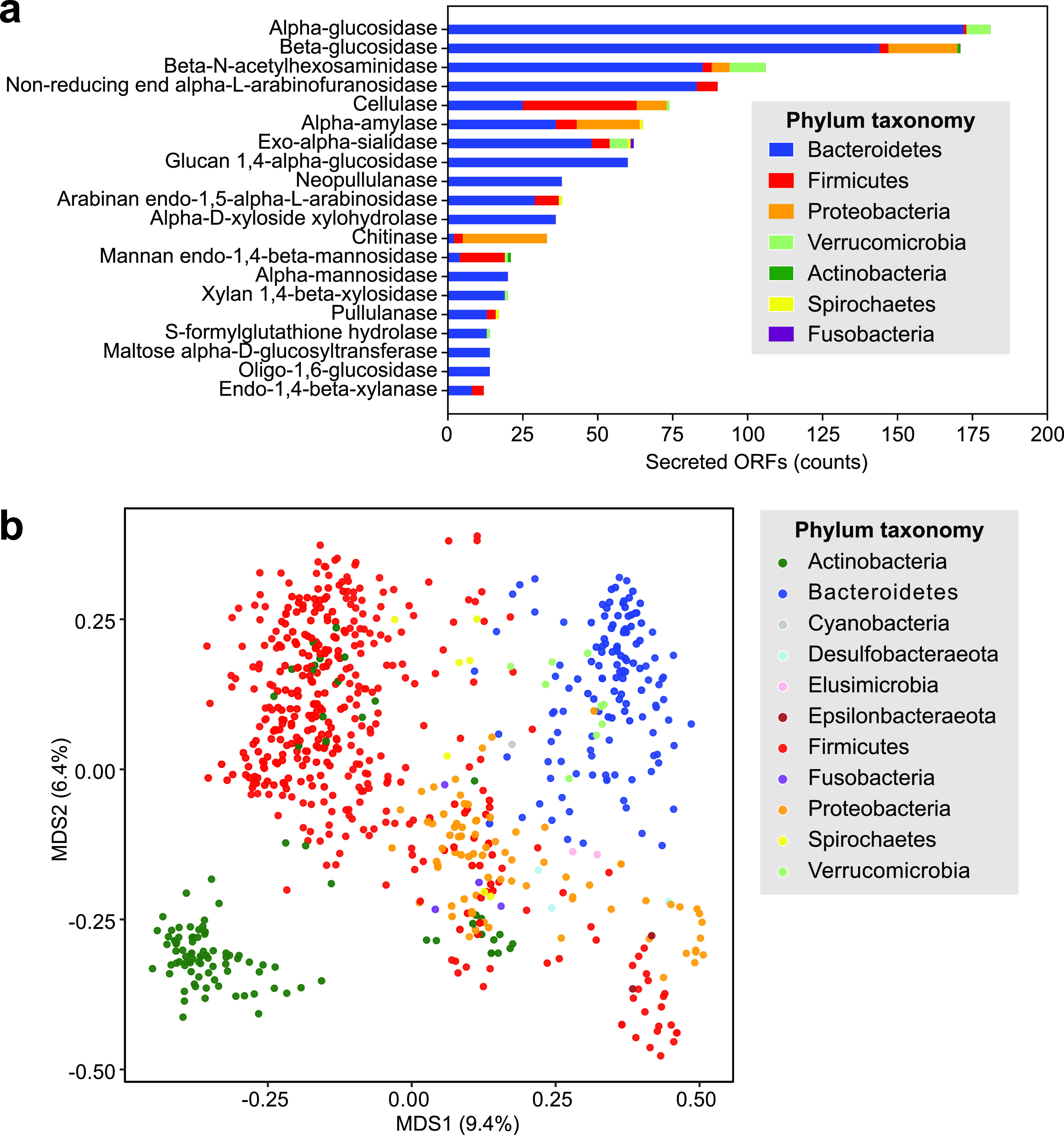
Carbohydrate degradation in the human gut metasecretome. (a) Most abundant secreted CAZyme families in the gut, colored by phylum. (b) Principal-coordinates analysis of representative MAGs secreted and nonsecreted with CAZyme abundance.

Since the gut microbiome contains a wealth of CAZymes and polysaccharide degradation plays a role in determining ecological niches within the gut, we wanted to define which gut bacterial CAZymes were likely to form part of the metasecretome. We used HMMER to annotate 1,911,738 ORFs from representative HGM MAGs (see Materials and Methods) with dbCAN CAZyme families, yielding 43,331 CAZyme annotations, 10.5% of which were secreted (Table S2). The most common CAZyme EC functions in the gut metasecretome included beta-*N*-acetylhexosaminidases, which are involved in degradation of chitin, intestinal mucosal glycans, and milk oligosaccharides (30–32), alpha-glucosidases, which are involved in the degradation of starch (33), and beta-glucosidases, which degrade cellulose (34, 35) (Fig. 3a). These results highlighted the complexity and abundance of gut bacterial enzymes dedicated to degrading human dietary substrates and host glycoproteins. When we compared the number of secreted CAZymes across different phyla, we found that *Bacteroidetes* and *Verrucomicrobia* secreted a higher percentage of their CAZymes than other gut phyla (Kruskal-Wallis H-test, *P* < 10^−3^; Mann-Whitney *U* test with Bonferroni correction, *P* < 10^−3^), in addition to encoding a large number of CAZymes (Fig. S2). To compare secretion of CAZyme families in gut bacterial MAGs, we performed principal-coordinates analysis (PCoA) (Fig. 3b). The principal coordinates were computed based on a matrix of the number of ORFs in each MAG that were annotated as part of a CAZyme family and whether these ORFs were annotated as secreted or not. We found that HGM bacterial phyla tended to cluster together, implying that a large part of the CAZyme repertoire is preserved at the phylum level in the human gut microbiome. However, *Verrucomicrobia* and *Bacteroidetes* MAGs tended to cluster closely together, suggesting that these MAGs have similar CAZyme repertoire features. To investigate this further, we examined the CAZyme abundance and secretion of MAGs in this *Bacteroidetes-Verrucomicrobia* (BV) cluster (Fig. S3). MAGs in the BV cluster tended to both encode and secrete a larger number of CAZyme families than did MAGs outside of this cluster. Many of the CAZymes unique to this cluster were predicted to be secreted and have been previously known to act upon animal carbohydrates (GH2, GH20, GH29, GH33, GH43, GH84, GH92, GH95, and GH109), plant cell wall carbohydrates (GH2, GH29, GH31, GH51, GH95, and GH127), or starch or glycogen (GH13) (6, 36). While some of these glycoside hydrolases have been previously reported in *Akkermansia municiphila*, other less-well-studied HGM *Verrucomicrobia* included in this study have not been previously reported to encode these CAZyme families. For instance, we identified several ORFs from *Opitutales* and UBA8416 MAGs as secretors of GH109 (data not shown), a CAZyme family with reported mucin-degrading abilities (37). However, these putative GH109 proteins had no close BLASTp matches and thus may represent novel degradative abilities in understudied members of the human gut *Verrucomicrobia*.

10.1128/msystems.00717-22.2 FIG S2 (a) Number of CAZyme ORFs in each HGM MAG. (b) Percentages of secreted CAZyme ORFs in each HGM MAG, ordered by average value of each phylum. Numbers of representative MAGs that were annotated in each phylum are shown above each plot. Download FIG S2, EPS file, 0.9 MB. Copyright © 2022 Velez-Cortes and Wang. 2022 Velez-Cortes and Wang. https://creativecommons.org/licenses/by/4.0/ This content is distributed under the terms of the Creative Commons Attribution 4.0 International license.

10.1128/msystems.00717-22.3 FIG S3 CAZyme family secretion in *Bacteroidetes*-*Verrucomicrobia* (BV) MAGs and non-BV MAGs. The percentages of secreted ORFs per MAG were averaged, with a pseudovalue of −25 for MAGs not encoding the CAZyme family. The table on the right shows broad substrate specificity for each CAZyme family (black = presence, white = absence). *, the GH144 family has beta-1,2-glucan activity. Download FIG S3, EPS file, 2.5 MB. Copyright © 2022 Velez-Cortes and Wang. 2022 Velez-Cortes and Wang. https://creativecommons.org/licenses/by/4.0/ This content is distributed under the terms of the Creative Commons Attribution 4.0 International license.

10.1128/msystems.00717-22.9 TABLE S2 CAZyme family annotations for HGM representative MAG ORFs, in Excel format. Download Table S2, XLSX file, 3.9 MB. Copyright © 2022 Velez-Cortes and Wang. 2022 Velez-Cortes and Wang. https://creativecommons.org/licenses/by/4.0/ This content is distributed under the terms of the Creative Commons Attribution 4.0 International license.

To understand the differences in secreted CAZyme repertoire in HGM bacterial families, we calculated the percentage of CAZymes secreted by each MAG in each CAZyme family in the HGM (Fig. S4). Most CAZyme families in the HGM are glycoside hydrolases and polysaccharide lyases, so we focused our analysis on these degradative enzymes. MAGs from the same phylum tended to cluster together, with some proteobacterial MAGs and some for *Firmicutes* as the exceptions. Verrucomicrobial MAGs tended to cluster closely with *Bacteroidetes*, suggesting a shared repertoire of CAZymes and thus similar glycan degradative capacities. Several glycoside hydrolases were more likely to be secreted in *Bacteroidetes* MAGs than in other phyla, including plant cell wall hydrolases (GH2, GH3, GH5, GH29, GH31, GH36, GH43, GH51, and GH127), peptidoglycan hydrolases (GH23, GH25, and GH73), sucrose or fructan hydrolases (GH32), and animal carbohydrate hydrolases (GH2, GH3, and GH29). However, these GHs were still encoded in the MAGs of most phyla in the data set, which suggested that they either fulfill other functions inside of the cell, or are exported via another route than the Sec pathway, or contain signal peptides that are not recognized by SignalP. GH1 and GH4 were two CAZymes that were encoded in most gut MAGs and not secreted, but they were also conspicuously not found in *Bacteroidetes* MAGs, despite being found in most other MAGs in the HGM, mainly *Firmicutes*. GH1 is a glycoside hydrolase family with beta-galactosidase, beta-glucosidase, and mannosidase activities. Some *Bacteroidetes* from nongut environments possess GH1, but thus far the only gut *Bacteroidetes* strain reported to possess GH1 is a ruminal strain of *Bacteroides* (38). GH4 has been mostly characterized in soil bacteria and participates in the degradation of raffinose, a plant-derived polysaccharide (39, 40). One of the few glycoside hydrolases that was more frequently secreted in *Firmicutes* than in *Bacteroidetes* was GH18, a CAZyme family that includes chitinases (EC 3.2.1.14) and endo-β-*N*-acetylglucosaminidases (EC 3.2.1.96). This may be indicative of a specific niche occupied by some *Firmicutes* strains in the gut or an alternative export mechanism for GH18 enzymes in *Bacteroidetes*. Together, our analysis of the human gut metasecretome demonstrated the diversity and abundance of secreted enzymatic functions in the healthy human gut. We showed that gut phyla encode vastly different CAZyme repertoires, with the exception of *Bacteroidetes* and *Verrucomicrobia*, which suggests an overlap in secreted degradative abilities among these two members of the gut microbiome. Finally, we identified novel putative glycosidase hydrolase families in verrucomicrobial MAGs.

10.1128/msystems.00717-22.4 FIG S4 CAZyme family secretion distribution across the HGM representative MAGs. Data show the percentages of ORFs in CAZyme families that are secreted in each representative MAG, with broad substrate categories for each CAZyme family. MAGs were clustered with a Euclidean metric, and CAZyme families were sorted by target substrate class. Download FIG S4, TIF file, 2.6 MB. Copyright © 2022 Velez-Cortes and Wang. 2022 Velez-Cortes and Wang. https://creativecommons.org/licenses/by/4.0/ This content is distributed under the terms of the Creative Commons Attribution 4.0 International license.

### Mapping the GI biogeography of secreted proteins.

To establish the general biogeography of secreted bacterial proteins in the gut, we aligned publicly available metagenomic reads from endoscopic and stool samples (41) to secreted ORFs predicted from a set of representative HGM MAGs by using Bowtie2 (see Materials and Methods) (Fig. S5a). Quality filtering of the metagenomic reads showed a high, albeit variable, sequencing depth across the human GI (Fig. S5b), and after mapping we were able to obtain reasonable coverage of HGM MAGs (Fig. S5c), which implied that our metasecretome ORFs were detectable in metagenomic GI reads.

10.1128/msystems.00717-22.5 FIG S5 (a) Pipeline used to map metagenomic reads from endoscopic samples against human gut MAGs. This figure was created using BioRender. (b) Number of metagenomic reads remaining after quality control filtering of endoscopic gut and stool samples. (c) Coverage of human gut MAGs from metagenomic reads from endoscopic samples along the GI tract. Download FIG S5, TIF file, 2.7 MB. Copyright © 2022 Velez-Cortes and Wang. 2022 Velez-Cortes and Wang. https://creativecommons.org/licenses/by/4.0/ This content is distributed under the terms of the Creative Commons Attribution 4.0 International license.

**FIG 4:**
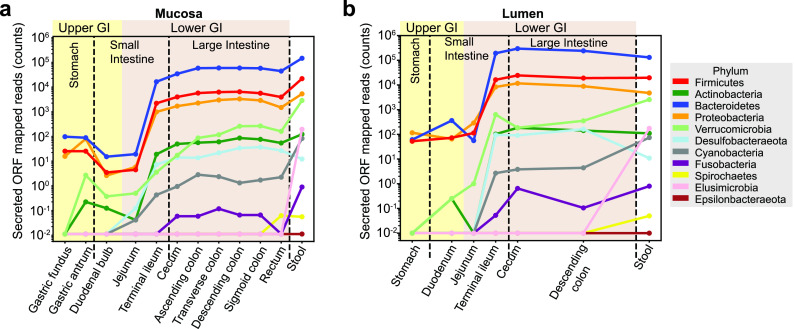
Biogeography of the gut metasecretome. (a) Reads mapping to secreted ORFs at the mucosa along the gastrointestinal tract. (b) Reads mapping to secreted ORFs in the lumen along the gastrointestinal tract.

By mapping metagenomic reads from endoscopic and stool samples to secreted ORFs from each phylum, we were able to estimate the contribution of each phylum to the metasecretome. We found that luminal samples mirrored stool samples more closely than mucosal samples and that samples taken at distal sites were more similar to stool samples than those from proximal sites (Fig. 4). This was similar to what was observed in the KEGG Orthology functional distribution of upper and lower GI in the original study (41). To some extent, this discrepancy arises from the varying taxonomic composition found across the GI tract, which reflects the need for different resource-harvesting abilities at each habitat within the GI tract. Across the entire GI tract, *Bacteroidetes*, *Firmicutes*, and *Proteobacteria* tend to encode the highest number of secretome ORFs of all gut phyla included in this analysis, with *Bacteroidetes* accounting for the highest number of mapped reads to secreted ORFs in the lower GI. *Bacteroidetes* tended to have more reads mapping to secreted ORFs in luminal samples than in mucosal samples, especially in the distal small intestine and proximal large intestine. This finding could be due to the diversity of dietary substrates available for degradation present in the luminal compartment. Based on the number of secretome reads mapped against the GI metagenomic samples, we determined that (i) the predicted metasecretome proteins were detectable throughout the GI tract, (ii) both mucosal and luminal compartments were abundant in secreted proteins, particularly in the lower GI, and (iii) secreted proteins from *Bacteroidetes* dominated the lower GI.

To determine which secreted protein functions were most abundant across different sublocalizations of the GI tract, we mapped GI endoscopic reads to ORFs from each EC function in the metasecretome (Table S3). We calculated a modified reads per kilobase per million reads (RPKM) metric for each EC function based on the mean nucleotide length of ORFs annotated with the EC function (see Materials and Methods). Most of the upper GI tract samples had little to no signal, which was likely due to (i) lower sequencing depth of upper GI tract samples (Fig. S5b) and (ii) lower overlap between HGM MAGs (15) derived from stool bacteria and bacteria residing in the upper GI tract (Fig. S5c). We focused on EC functions with pseudo-RPKM values higher than 50 in at least one GI site in order to reduce effects from noise and analyze more-prevalent ECs. We found that two EC functions, beta-galactosidase (EC 3.2.1.23) and alpha-l-fucosidase (EC 3.2.1.51), had the highest relative abundances in luminal and mucosal lower gastrointestinal tract samples (Fig. S6). While nearly 70% of the population is lactose intolerant (42), generally the Western human diet is high in lactose, which is a substrate for beta-galactosidases (3), and previous studies have noted the presence of this enzyme in *Bifidobacteria* in the gut microbiome (43).

10.1128/msystems.00717-22.6 FIG S6 (a) Pseudo-RPKMs for EC functions in GI tract and stool samples for all ECs that had over 50 RPKM in at least 1 site, in luminal and mucosal endoscopic and stool samples. (b) Corresponding EC descriptions. Download FIG S6, EPS file, 2.7 MB. Copyright © 2022 Velez-Cortes and Wang. 2022 Velez-Cortes and Wang. https://creativecommons.org/licenses/by/4.0/ This content is distributed under the terms of the Creative Commons Attribution 4.0 International license.

10.1128/msystems.00717-22.10 TABLE S3 Pseudo-RPKM counts of HGM secreted proteins mapping to GI site, in Excel format. Download Table S3, XLSX file, 0.1 MB. Copyright © 2022 Velez-Cortes and Wang. 2022 Velez-Cortes and Wang. https://creativecommons.org/licenses/by/4.0/ This content is distributed under the terms of the Creative Commons Attribution 4.0 International license.

**FIG 5:**
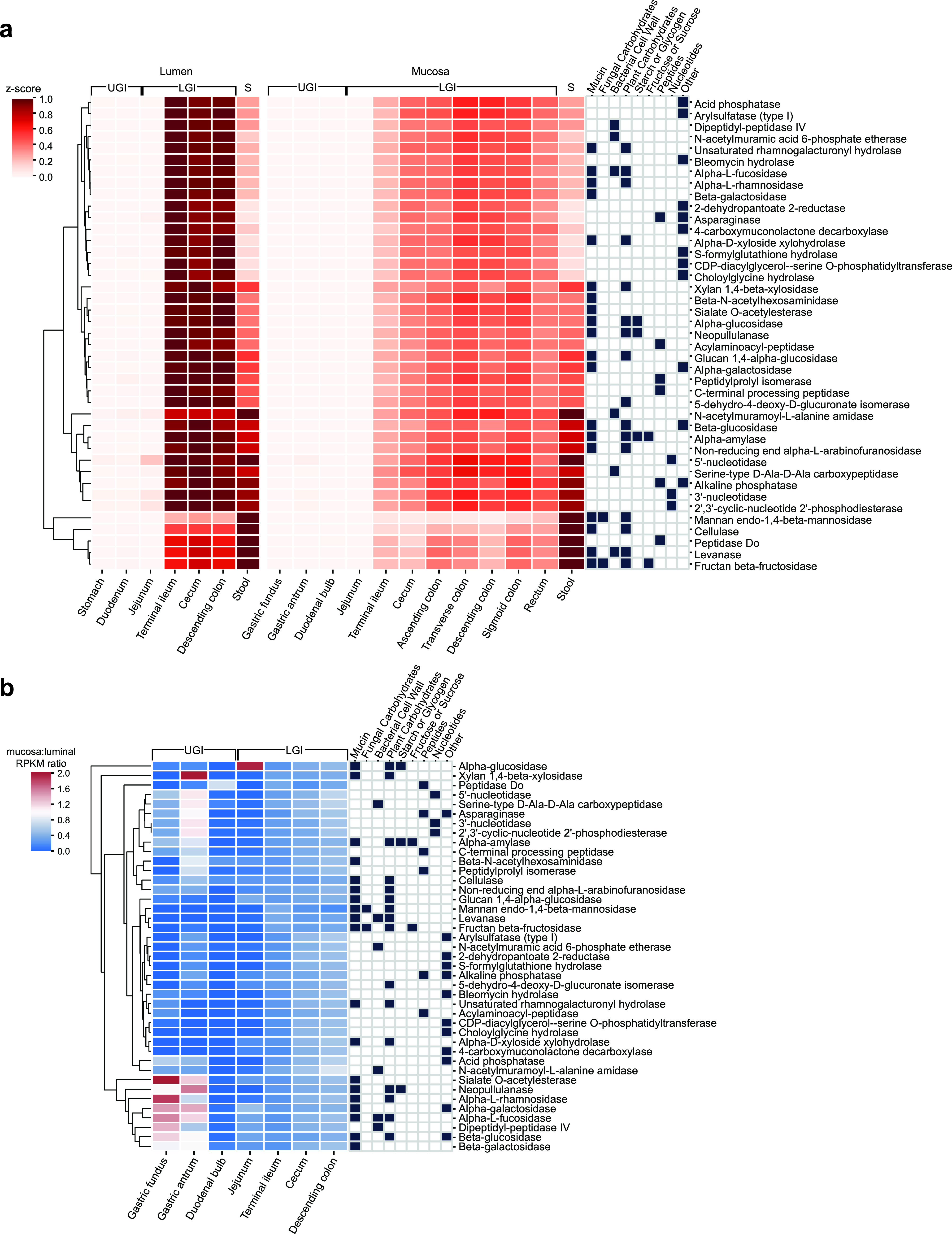
Biogeography of gut microbiome secreted functions. (a) *Z*-score of RPKM abundance of EC categories across gastrointestinal luminal and mucosal samples, with stool sample repeated for ease of comparison. (b) Ratio of mucosal to luminal RPKM abundance of EC categories across the gastrointestinal tract. Sample RPKM labels: UGI, upper gastrointestinal tract; LGI, lower gastrointestinal tract; S, stool.

To compare the abundance of each secreted enzyme across sites, we normalized the RPKM values of each GI site against the highest RPKM for that enzyme, generating a *z*-score for each EC and GI site. Most of the secreted proteins that we were able to map were more abundant in lower GI samples than in stool, including numerous glycosidases (EC 3.2.1.x) and 2-dehydropantoate 2-reductase (EC 1.1.1.169) (Fig. 5a and Fig. S7a). The most abundant secreted EC functions in the upper GI tract were 5′-nucleotidases and 3′-nucleotidases. There were also several secreted glycosidases, such as cellulose and mannan endo-1,4-beta-mannosidase, as well as a serine endopeptidase, that were overrepresented in stool samples relative to the rest of the GI tract. Generally, the lower GI tract was enriched in secreted proteins, which is expected since *Bacteroidetes* tend to reside in the colon (9) and are major contributors to the metasecretome.

10.1128/msystems.00717-22.7 FIG S7 (a) EC function abundance across GI tract and stool samples, represented by RPKM values normalized to the highest RPKM in EC across all luminal and mucosal samples, as determined for luminal and mucosal endoscopic samples. (b) Ratio of luminal versus mucosal samples across the GI tract. Download FIG S7, EPS file, 1.8 MB. Copyright © 2022 Velez-Cortes and Wang. 2022 Velez-Cortes and Wang. https://creativecommons.org/licenses/by/4.0/ This content is distributed under the terms of the Creative Commons Attribution 4.0 International license.

The luminal and mucosal compartments of the GI contain differing substrates that microbes can feed on, with higher concentrations of mucin and other host glycans at the mucosa and fiber and starches from digesta in the lumen. We posited that these differences in substrates would result in secreted degradative proteins at different parts of the intestine. To identify secreted EC functions that were specific to mucosal or luminal sites of the GI tract, we took the ratio of RPKM in mucosal sites and luminal sites (Fig. 5b and Fig. S7b). We identified glycosidases that were overrepresented in the stomach mucosa, in particular, xylan 1,4-beta-xylosidase, sialase *O*-acetlyesterase, neopullulanase, alpha-l-rhamnosidase, alpha-galactosidase, alpha-l-fucosidase, dipeptidyl-peptidase IV, beta-glucosidase, and beta-galactosidase. In general, most putative secreted EC functions were enriched in the luminal areas of the GI tract. However, the distribution of secreted EC functions became more evenly distributed between luminal and mucosal compartments as we approached the distal end of the GI tract. Since the mucus layer becomes thicker in the colon (9), the mucosa can harbor more bacterial metabolic activity in the form of mucin degradation (44) and may support subsequent cycles of cross-feeding interactions.

## DISCUSSION

Here, we have presented an extensive data set of human gut secreted proteins and an analysis of their main functions and distribution in the gut. When we analyzed the phylogenetic distribution of secreted proteins, we found that the number of secreted proteins encoded in different phyla varied widely, with *Bacteroidetes* being the main contributor to the gut metasecretome. Moreover, *Verrucomicrobia* also appeared to secrete a notable portion of its proteome. We performed the most extensive comparative analysis of secreted CAZymes in the gut to date and showed that *Verrucomicrobia* and *Bacteroidetes* encode similar secreted CAZyme repertoires, which hints at similar glycan degradation abilities. Further study on cultured isolates is necessary to verify the degradative abilities that are encoded by these predicted secreted CAZymes. A previous study found evidence of *Bacteroidetes*-*Verrucomicrobia* horizontal gene transfer in the human gut microbiome, and CAZymes have been shown to be part of the mobilome (45), but further research is needed to determine whether secreted CAZymes are being shared between *Verrucomicrobia* and *Bacteroidetes*.

In this work, we found that *Bacteroidetes* and *Verrucomicrobia* tend to export a substantial fraction of their proteome. A large proportion of the secretome of these two phyla is dedicated to CAZymes that participate in the breakdown of complex carbohydrates, which has been shown previously in certain members of these phyla in the gut (3, 5). Our observations reinforced a view that places most *Bacteroidetes* and *Verrucomicrobia* in the highest trophic level of the gut, where they can utilize dietary fibers and mucin directly, while other phyla benefit from the breakdown products derived from these primary degradation reactions (46, 47). We also observed that secreted animal carbohydrate hydrolases were present in higher numbers in *Akkermansiaceae* and *Bacteroidaceae* among other *Verrucomicrobia* and *Bacteroidetes* families compared to other gut phyla (data not shown), which further suggested that strains from these families are specialized in host glycan degradation. Development of methods for the study of complex communities (48, 49) as well as more high-throughput assays that can characterize CAZyme substrate specificity will be helpful in elucidating the ecological interactions and dynamics of gut bacteria.

We have taken care to impose several filters on our secreted protein prediction; however, signal peptide prediction is not always accurate, although algorithms have improved over the last decade. The genomes used to train these algorithms reflect the availability of current data, which is biased toward more well-researched phyla, such as *Proteobacteria*. We also expect that a portion of the predicted secreted proteins are periplasmic. Since some of these periplasmic proteins may become public goods via outer membrane vesicles (50), we decided to not filter them. Because of computational resource limitations, we focused our functional analysis of secreted proteins on one MAG per strain-level OTU. We also clustered protein sequences and removed proteins that did not cluster with at least 4 other proteins in the data set. This underestimated the true diversity of the secretome, which likely varied highly at the strain level. However, we expect this approach still leaves us with a comprehensive catalog of the gut microbiome metasecretome. Finally, while many CAZymes we found are associated with breakdown of mucin or dietary fiber or other sources of nourishment for the gut microbiome, some CAZymes we identified in the gut are not unique to the gut and are involved with energy production (GH1, GH13, GH31, GH32, and GH38) or peptidoglycan breakdown (GH23, GH25, and GH73) (6).

To our knowledge, this is the first study to map the bacterial secretome across the GI tract, in which we validated the prevalence of the predicted metasecretome proteins and identified functional enrichment in different gut habitats. We observed that many secreted glycosidases are enriched in the luminal lower GI tract and are underrepresented in stool samples. This underscores the importance of expanding our studies beyond stool isolates and ensure the representation of bacteria unique to the GI tract in culturomics efforts. Given that the gut presents a unique habitat for cooperativity and competition among bacteria (12) with opportunities for the evolution of secreted proteins with undiscovered degradative abilities (32), the present study shows the vastness of secreted proteins in the gut has barely been uncovered and represents an opportunity to discover new interactions among bacteria and between humans and their gut microbiome.

## MATERIALS AND METHODS

### MAGs and ORF annotation.

We used the high-quality Human Gut Metagenome (HGM) MAGs reported by Nayfach et al. (15). We selected MAGs that were in OTUs that were classified as Bacterial by Nayfach using GTDB-Tk (51). We annotated each MAG using Prodigal version 2.6.3 (17) to identify ORFs by using modified settings that allowed for smaller ORF discovery (52). Briefly, we modified prodigal source code so that we could find smaller genes; specifically, we changed the MIN_GENE parameter in Prodigal-2.6.3/node·h to 15, so that we could identify ORFs that had 15 nucleotides or more. To ensure the MAGs we were using were of sufficient quality, we required a minimum of 482 ORFs per MAG, since that is the number of genes in the smallest known bacterial species, Mycoplasma genitalium (53). We selected representative gut MAGs by taking one MAG at random from each species-level OTU in the HGM high-quality MAG data set, resulting in 765 representative gut bacterial MAGs.

ORFs from representative MAGs were annotated with HMMER (54) using the dbCAN CAZyme database (55) and eggNOG v5.0 database (26). HMMER search criteria for CAZyme identification included 0.35 minimum coverage of the CAZyme and a minimum e-value of 1e−15, which were enforced using the hmmscan-parser tool from dbCAN. eggNOG search results were required to have a minimum e-value of 0.001. The CAZyme heatmap was clustered using seaborn with Euclidean metric and single method. CAZyme families were removed from the heatmap if present in only one representative MAG. Broad substrates were obtained using the supplementary table from Cantarel et al. (6), in addition to literature searches using CAZydb.

### Phylogenetic analysis.

To construct a phylogenetic tree of all the families of interest in the Nayfach data, we used the same method used by Nayfach but on a select set of representative MAGs, one from each bacterial OTU, totaling 765 MAGs. In brief, GTDB-Tk was used with the classify_wf workflow to call the marker protein sequences from MAGs using Prodigal and HMMER, aligned the concatenated marker sequences, and used pplacer to construct a maximum-likelihood tree. This tree was plotted using iqtree version 1.6.12 with the following server command: iqtree -s gtdbtk.bac120.user_msa.fasta -st AA -m MFP -nt 4.

### Clustering and SignalP secretion tag prediction.

We clustered human gut metagenome ORFs with USEARCH (18) at 95% amino acid sequence identity. Representative sequences of clusters with more than 5 sequences were annotated using SignalP 5.0 (19) to predict secretion tags. Sequences with a tag corresponding to type 1 secretion or TAT secretion were considered “secreted,” and those for lipoprotein or no secretion tag were considered “not secreted.” Protein sequences were further annotated using TMHMM (22) to determine whether they contained transmembrane domains. Proteins that contained transmembrane domains were classified as not secreted.

### Principal-coordinates analysis of CAZymes in representative human gut MAGs.

An in-house R script (56) was used to create a matrix of counts of ORFs present in each CAZyme family from CAZydb annotations of the representative human gut MAGs data set. If a CAZyme family had ORFs that were secreted and nonsecreted, we counted these as two separate CAZyme families. We then calculated a distance matrix based on the Spearman correlation of the CAZyme family counts.

### Mapping of secreted proteins to GI tract.

We used bowtie2 version 2.4.2 (57) to align Elinav metagenomics reads from endoscopic and stool samples to a set of representative gut MAGs. First, we applied a similar quality-filtering method to that used by Elinav et al. (41) for their samples. That is, we used trimmomatic version 0.39 (58) to perform adapter trimming using the following command: trimmomatic PE -validatePairs -threads 2 -phred33 input_readsQC/eachSample_R1_001.fastq.gz input_readsQC/eachSample_R2_001.fastq.gz eachSample_R1_paired.fq.gz eachSample_R1_unpaired.fq.gz eachSample_R2_paired.fq.gz eachSample_R2_unpaired.fq.gz ILLUMINACLIP: TruSeq3-PE.fa:2:30:10 LEADING:3 TRAILING:3 MINLEN:50.

Then, we performed filtering of human reads from the samples using bowtie2 against the bowtie2 index for the human genome reference Hg19. We counted unique reads mapping to secreted ORFs from each phylum and for each Enzyme Commission functional annotation using an in-house script. We calculated a pseudo-RPKM value for each sample using the mean nucleotide lengths of ORFs in representative HGM MAGs that were annotated with a particular EC function. Samples taken from the same GI site were averaged. We included in the heatmaps only EC categories that had a minimum value greater than 50 RPKM for at least one GI site. To normalize all EC function RPKMs, we divided RPKM values in an EC function by the maximum RPKM for that EC function across all GI tract and stool samples.

### Statistical analysis.

Python package SciPy (59) was used to perform a Mann-Whitney *U* test to determine *P* values for the differences in protein length between secreted and nonsecreted ORFs. The same package was used to perform a Kruskal-Wallis test and *post hoc* Mann-Whitney *U* tests with Bonferroni corrections to determine differences in biosynthetic costs from each major phylum and differences in percentages of proteins secreted by each major phylum and of CAZymes secreted by each major phylum. MAGs were considered to be major gut phyla if there were over 50 MAGs in the data set.

### Code availability.

Metasecretome prediction scripts can be accessed at https://github.com/fgv2104/gut_metasecretome.

### Data availability.

Predicted HGM secreted protein sequences are available for download as HGM_secreted_orfs.faa.gz from https://github.com/fgv2104/gut_metasecretome.
