# Supplementary figures and images for: Characterization and Spatial Mapping of the Human Gut Metasecretome
Source: mSystems. 2022 Dec 5;7(6):e00717-22. doi: 10.1128/msystems.00717-22 (PMC9765747; doi:10.1128/msystems.00717-22)

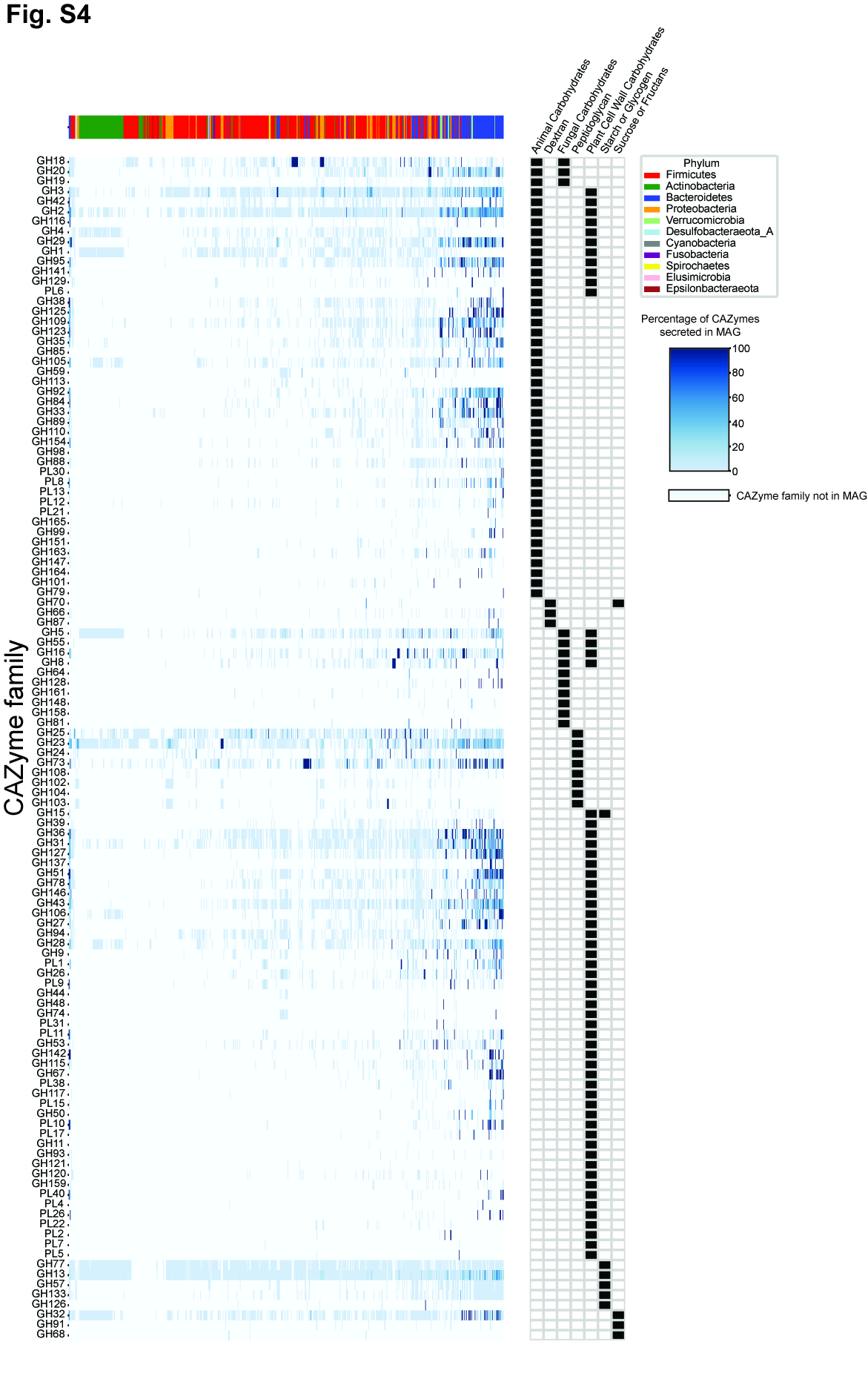

Supplement: FIG S4 [file msystems.00717-22-sf004.tif]

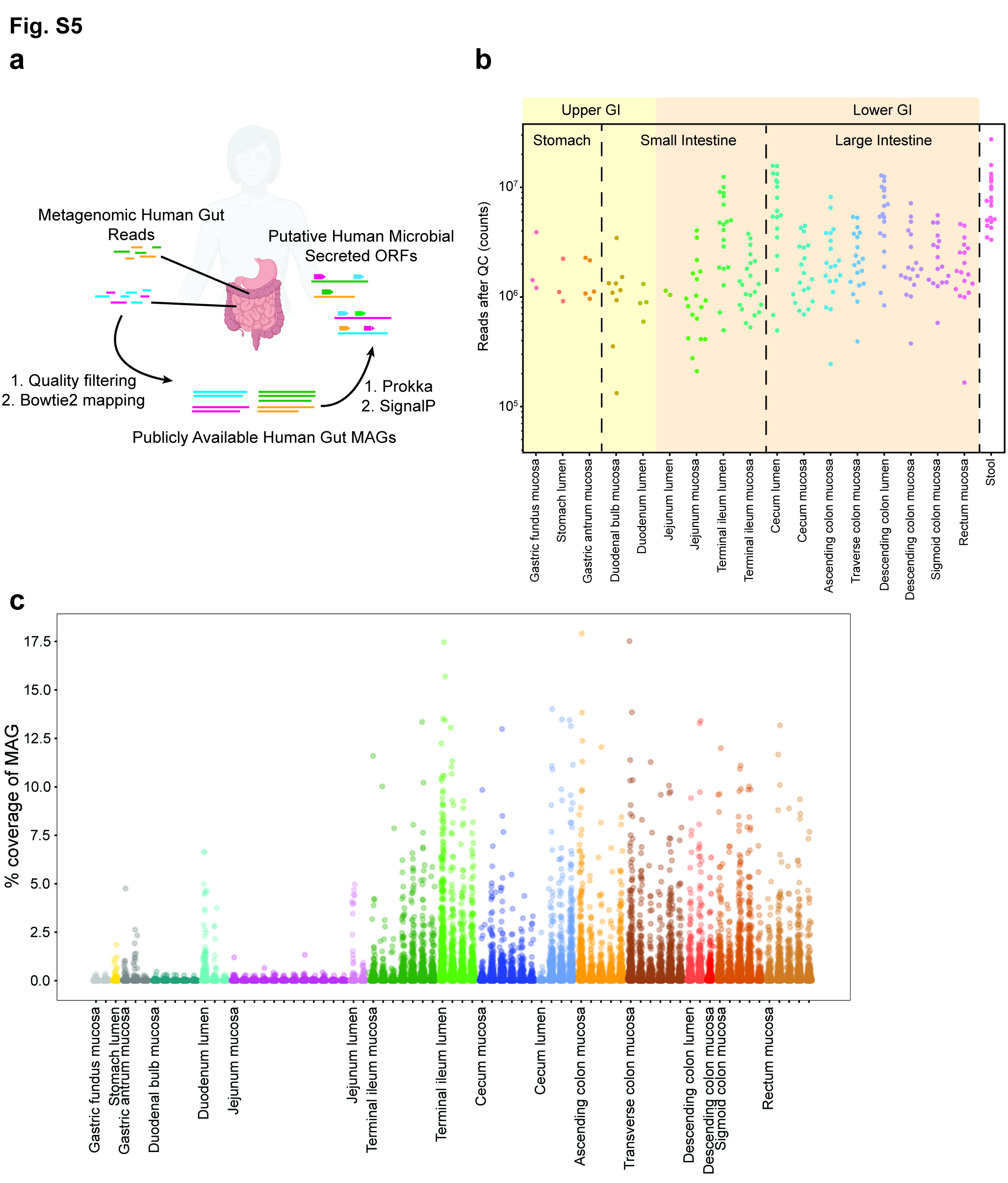

Supplement: FIG S5 [file msystems.00717-22-sf005.tif]
